# Adsorption of different anionic and cationic dyes by hybrid nanocomposites of carbon nanotube and graphene materials over UiO-66

**DOI:** 10.1038/s41598-022-24891-2

**Published:** 2022-11-27

**Authors:** Mohammadreza Athari, Moslem Fattahi, Mohammadreza Khosravi-Nikou, Aliasghar Hajhariri

**Affiliations:** 1 Department of Chemical Engineering, Abadan Faculty of Petroleum Engineering, Petroleum University of Technology, Abadan, Iran; 2 Department of Gas Engineering, Ahvaz Faculty of Petroleum, Petroleum University of Technology, Ahvaz, Iran; 3 Fakultät für Verfahrens- und Systemtechnik (FVST), Otto von Guericke Universität (OVGU), Magdeburg, Germany

**Keywords:** Chemistry, Chemical engineering, Physical chemistry, Surface chemistry, Chemical synthesis

## Abstract

Amongst many chemical pollutants that cause environmental pollution, the presence of organic dyes in water resources can cause substantial health issues. Thus, owing to their mutagenicity and their adverse effects on human health, environment, and animals, they must be removed from industrial wastewater. In this study, UiO-66 metal–organic framework, as well as composite nanoparticles with carbonaceous materials such as MWCNTs-COOH and graphene oxide (GO) with different molar ratios (2.9 and 5.8 wt.%), were synthesized through solvothermal method since carbonaceous materials are an emerging material that demonstrates improvement in the properties of adsorbents. Then, the synthesized materials were utilized as a solid adsorbent for removing four different dyes including; anionic methyl red (MR), anionic methyl orange (MO), cationic methylene blue (MB), and cationic malachite green (MG) prepared from distilled water. The properties of prepared adsorbents were characterized via X-ray diffraction (XRD), Fourier transform infrared spectroscopy (FTIR), field emission scanning electron microscopy (FESEM), Photoluminescence spectroscopy (PL), Brunauer–Emmett–Teller (BET), as well as surface area analyzer and energy dispersive spectroscopy (EDS-MAP). Further, the influences of various factors including initial concentrations of the dyes and adsorption process time on adsorption of dyes were investigated. Adsorption experiments indicated that synthesized adsorbents exhibited the highest adsorption efficiency towards MR and MO dyes. Moreover, the experimental adsorption results revealed that MWCNTs-UiO-66 nanocomposites could adsorb 98% of MR and MO as well as 72% of MB and 46% of MG. Furthermore, the kinetic and stability of the materials over time were investigated. To reach a clear picture, adsorption experiments demonstrated that the amount of dye uptake on adsorbents was enhanced by increasing the contact time as well as uptake of materials with time were stable for both cationic and anionic dyes. The MR, MO, and MB adsorption isotherms were fitted with the Langmuir and Freundlich models. The Langmuir showed the highest agreement in these dyes and MWCNTs-UiO-66 (2.9 and 5.8 wt.%) exhibited a maximum adsorption capacity of 105.26 mg/g for MR, while the MG isotherm was in line with the Langmuir model.

## Introduction

Nowadays, many chemical pollutants such as toxic heavy metals, aromatic compounds, pharmaceutical products, especially organic dyes, and other pollutants in large quantities enter the environment and water resources, with increasing production worldwide^1^. Organic dyes are commonly utilized in textile, oil, gas, petrochemical, paint, rubber, printing, cosmetics, plastic, photographic, paper, and other industries^2,3^. Organic dyes are toxic, non-biodegradable, carcinogenic, and mutagenic, which can damage the environment, human health, animal and aquatic life. The organic dyes are stable against sunlight thereby acting as oxidizing agents^4^. Thus, these chemical pollutants that have irreversible and adverse effects must be removed from contaminated water by using an effective method. Numerous methods have been reported to remove organic dye from industrial wastewater including photocatalytic degradation, electron Fenton, membrane separation, ion exchange, and adsorption^5^. Among these various techniques, adsorption is considered as an effective and economical technique for dye removal owing to its outstanding benefits such as high efficiency, simple operation, low cost, shorter process time, and low energy consumption as well as do not produce secondary pollutants^6–8^. A good adsorbent is expected to possess large specific surface area, fast adsorption rate, simple preparation, high porosity and pore volume, less cost, high adsorption capacity, good thermal and mechanical properties as well as chemical stability and good selectivity^9–11^. Owing to different physical and chemical properties of cationic and anionic dyes, finding suitable adsorbents for dye removal with high efficiency and fast adsorption kinetics has become an area of interest.

Over the last two decades, utilization of different types of porous materials such as porous organic polymers (POP), metal–organic frameworks (MOFs), activated carbons, zeolite, hydrogels, functionalized mesoporous silica, graphene oxide, and carbon nanotubes (CNTs) has been studied for dye removal^4,11^. Among these, the MOFs as a new class of highly porous and crystalline materials, constructed from the coordination of metal clusters and organic ligands, exhibit many interesting characteristics such as large capacity for the adsorption, tunable pore walls, chemical stability, and controllable porosity. The variety of MOFs topologies and tunable chemical functionalities make MOFs attractive for various applications such as adsorption/storage of carbon dioxide, hydrogen storage, adsorption of vapors, separation of chemicals, drug delivery/biomedicine, polymerization, magnetism, catalysis, and luminescence^12^. Thus, these are good candidates for removal of aqueous hazardous materials. In spite of good features, most of these materials are unstable in water and also subject to degradation through ligand displacement or hydrolysis^13,14^. Among various MOFs, UiO-66 (abbreviation of the University of Oslo) which possesses exceptional chemical, thermal and mechanical stability (attributing to a high coordination number (12) of the [Zr_6_(m3-O)_4_(m3-OH)_4_] core with bidentate benzene-1,4- dicarboxylic acid (BDC) ligand) and its composite nanoparticles display high stability in aqueous solutions. The Zr-based MOFs, especially UiO-66 have displayed special strength against water because of the strong Zr-O bond and a specific geometry that hinders the inclusion of water molecules to mitigate the hydrolysis reactions^10,15,16^. Carbon-based MOFs composites for example graphene-MOF and carbon nanotubes (CNTs)-MOF are attractive adsorbents. Heterojunction of CNTs with different materials such as MOF shows excellent capability to remove organic pollutants. The CNTs have significant mechanical, and thermal properties that can be a good adsorbent and dispersing agent in dye removal due to the high specific surface. Thus, various amounts of this material were added in the preparation of composites to investigate adsorption capacity^17–20^. Since the first studies on the application of MOF in pollutants degradation (catalytic degradation of phenol by MOF-5 in 2007^21^), especially the adsorption of pollutants from water (adsorptive removal of methyl orange (MO) by MIL-53, and MIL-101(Cr) in 2010^22^), remarkable studies have been accomplished on the usage of MOFs in wastewater treatment. The application of UiO-66 and its composite nanoparticles has been well reported in the literature. For example, Molavi et al. synthesized UiO-66 in the presence of thermally oxidized nanodiamond (OND) for ultrafast and simultaneous removal of anionic and cationic dyes. OND-UiO-66 exhibited a maximum adsorption capacity of 556 mg/g for MR which was far higher than that of single UiO-66 and OND^3^. Furthermore, solvothermally synthesized N_2_-UiO-66 for enhanced and selective adsorption of cationic dyes was reported by Tambat et al. They observed enhanced adsorption capacity with increase in the initial dye concentration^23^. Sang et al. studied interfacial growth of metal–organic framework on carboxyl functionalized carbon nanotube for efficient dye adsorption and separation. This research demonstrated that the interfacial incorporation of CNTs-COOH can improve physical the morphology of UiO-66-NH_2_ as well as improve MO dye adsorption capacity plus selectivity^24^. Furthermore, superior chemical stability of UiO-66 metal–organic frameworks (MOFs) for selective dye adsorption was reported by Ahmadijokani et al. where pristine UiO-66 and its solvent aged samples showed good selectivity for anionic dyes against cationic dyes^4^. In another study, Tambat et al. reported solvothermal synthesis of NH_2_-UiO-66 and its application for adsorptive removal of safranin dye, where the maximum adsorption capacity of the MOF was observed to be 390 mg/g^25^. Elsewhere, He et al. studied adsorptive removal of Rhodamine B on UiO-66 with the maximum dye adsorption capacity observed about 75 mg/g^26^. The above studies suggest that upon adding a suitable functional group to pure MOF (UiO-66), selective adsorption towards a particular type of target compound can be achieved. In another study, Abdi et al. reported amine-functionalized Zr-MOF/CNTs nanocomposite as an efficient and reusable photocatalyst for removing organic contaminants, where UiO-66-NH_2_@CNT (3 wt%) exhibited the highest degradation efficiency of both dyes (100% for RhB and more than 93% for MO) in less than 30 min^17^.

The aim of this study is solvothermal synthesis of UiO-66 and its composite nanoparticles with carbonaceous materials such as MWCNTs-COOH and graphene oxide (GO) for adsorption of MG, MB cationic dyes as well as MR, MO anionic dyes from distilled water. For this purpose, various amount of carbonaceous material added for preparation of composites to investigate adsorption capacity. The influences of various factors were investigated including initial concentrations of the dyes and adsorption process time. Further, the two models of Langmuir and Freundlich isotherms were utilized for investigating the best isotherm for each dye.

## Materials and methods

### Materials

All chemicals including zirconium (IV) chloride (ZrCl_4_, 98%), N, N-dimethylformamide (DMF, 99%), terephthalic acid (H_2_BDC, 99%), chloroform (99%), MWCNTs-COOH (39 nm), graphene oxide (3–18 nm), methyl red (MR), methyl orange (MO), methylene blue (MB), and malachite green (MG) were purchased from Merck and Sigma Aldrich companies and utilized without further purification due to their analytical grade. Deionized (DI) water was employed to prepare all solutions.

### Characterization

To study the crystallography of the samples, X-pert Philips pw 1730 (Netherland) X-ray diffractometer operating at current of 30 mA and accelerating voltage of 40 kV, using Cu-Kα1 (wavelength of 1.54056 Å) radiation in scanning range of 2θ = 10°–70° was utilized at a scanning speed of 1 s/0.05◦. Fourier transform infrared (FTIR) spectroscopy analyses was conducted using THERMO AVATAR (USA) FT-IR Spectrometer with the wavelength range of 400–4000 cm^−1^. The surface morphology of synthesized nanohybrids was observed by using FESEM–MIRA3 TESCAN (Czech). N_2_-physisorption experiments were carried out at 77 K in using BELSORP MINI II (Japan) instrument. Photoluminescence (PL) spectra were evaluated using Varian Cary Eclipse Fluorescence Spectrophotometer.

### Synthesis of pure MOF nanoparticles

UiO-66 was synthesized through the procedure reported in references^3,25,27^. UiO-66 MOF and its composite nanomaterials were synthesized through solvothermal method. For synthesis of UiO-66, 0.53 g ZrCl_4_ as a metal source and 0.38 g terephthalic acid (H_2_BDC) as a linker were dissolved into 30 mL DMF under continuous stirring at 25 °C for 1 h to obtain a homogeneous solution. The solution was transferred to a Teflon autoclave and was maintained inside an oven at 120 °C for 1 day. The autoclave was cooled down to room temperature with UiO-66 nanoparticles collected through centrifugation at 8000 rpm for 10 min. The as-synthesized UiO-66 nanoparticles were washed three times with DMF via centrifugation method at 6000 rpm for 10 min and subsequently several times with chloroform by centrifugation method at 6000 rpm for 10 min followed by sonication for 10 min to enhance its crystallinity and remove unreacted reactants, and then solvent-exchanged by soaking for 5 days in chloroform. Next, the synthesized nanoparticles were heated in vacuum oven at 100 °C overnight. Finally, crystalline material of UiO-66 was obtained.

### Synthesis of MWCNTs-UiO-66 hybrid nanoparticles

MWCNTs-UiO-66 composites were synthesized by adding carboxylic acid functionalized CNTs (MWCNTs-COOH) powders into the synthesis system of UiO-66^24^. Briefly, for the synthesis of MWCNTs-UiO-66 nanoparticle, similar amounts of H_2_BDC and ZrCl_4_ utilized for synthesized pure UiO-66 were dissolved into DMF and stirred at 25 °C for 1 h to produce a clear solution. Then, MWCNTs-COOH was well-dispersed in the above precursor solution under sonication for 1 h. The mixture was transferred to a Teflon autoclave and was kept inside an oven at 120 °C for 1 day. After cooling, the as-synthesized composite was separated through centrifugation at 8000 rpm for 10 min and was then washed with DMF via centrifugation method at 6000 rpm for 10 min, and then washed with chloroform several times by centrifugation at 6000 rpm for 10 min under sonication for 10 min to remove the organic impurity. Similar to the procedure used for the synthesis of UiO-66, nanoparticles were solvent-exchanged by soaking in chloroform for 5 days. Finally, the obtained product was dried in vacuum oven at 100 °C overnight. The MWCNTs-COOH amounts added in the preparation of the composites were separately 2.9 and 5.8 wt.% of the initial material weight, which were named MWCNT1:UiO1 and MWCNT2:UiO1, respectively.

### Synthesis of graphene oxide-UiO-66 hybrid nanoparticles

The graphene oxide-UiO-66 nanoparticles were prepared by the same procedure; though same amount of graphene oxide was used instead of MWCNTs-COOH. The graphene oxide amounts added in the preparation of the composites were separately 1.5, 2.9, and 5.8 wt.% of the initial material weight, which were named Graphene1:UiO2; Graphene1:UiO1, and Graphene2:UiO1, respectively.

### Batch adsorption experiments

The adsorption capacities of UiO-66 and its composite nanoparticles were examined for four different dyes including anionic methyl red (MR) dye, anionic methyl orange (MO) dye, cationic Methylene blue (MB) dye, and cationic malachite green (MG) dye from distilled water. Solutions with different initial concentrations as C_0_ = 10 mg/L, 20 mg/L, and 40 mg/L were produced by adding dyes in distilled water. The optimal amount of adsorbent dose was considered equal to 0.5 g/L. In a typical experiment, 20 mg of adsorbents was added to a 40 mL of dye solution and the suspension was continuously stirred using a magnetic stirrer for 2 h at room temperature. After 1 h, Sampling of the dye solution was done and the adsorbent nanoparticles were collected from the dye solution using centrifugation at 8000 rpm for 10 min. Then, the dye concentrations were determined using the characteristic absorbance (617 nm for MG, 435 nm for MR, 464 nm for MO and 663 nm for MB) of the dye solutions using a UNICO 2800 UV/VIS spectrophotometer^3,25,28,29^. Then after another hour, re-sampling was carried out and the above steps were repeated. Up to 4 h, sampling of the dye solution was done every 1 h and the dye concentrations were determined. These steps were done separately for each of the dye solutions with different initial concentrations as C_0_ = 10 mg/L, 20 mg/L, and 40 mg/L to determine concentration of dye solutions.

The extent of dye uptake on the adsorbent at different times, t, is represented by q_t_ and calculated using the following mass balance equation:

1 $${\text{q}}_{\text{t}}= \frac{ \left({\text{C}}_{0}- {\text{C}}_{\text{t}}\right)\text{ V}}{\text{W}}$$

where, C_0_ is the initial concentration (in mg/L) of dye, C_t_ denotes the concentration (in mg/L) of dye at time, t, V and W represent the volume (in L) of the dye solution and the amount (in g) of adsorbent, respectively.

Further, equilibrium adsorption capacity, q_e_ (mg/g), was calculated by:

2 $${\text{q}}_{\text{e}}= \frac{ \left({\text{C}}_{0}- {\text{C}}_{\text{e}}\right)\text{ V}}{\text{W}}$$

where, C_e_ is the equilibrium concentration (in mg/L) of dye. All other quantities on the right side of the above equation have the same description as in Eq. (1)^23,25^.

## Results and discussion

### Characterization

**Figure 1:**
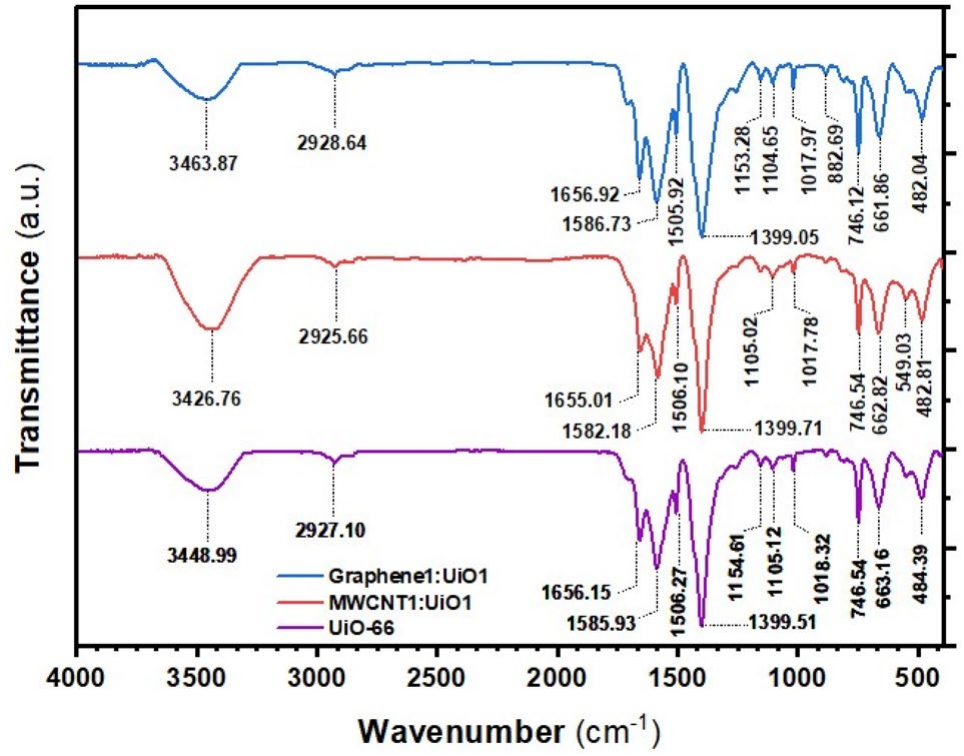
FTIR spectra of UiO-66, MWCNT1:UiO1 and Graphene1:UiO1.

Figure 1 displays the Fourier transform infrared spectroscopy (FTIR) spectra of the synthesized MOFs. All three synthesized adsorbents showed major peaks at 1655^−1^, 1585 cm^−1^, 1399 cm^−1^, 746 cm^−1^, and 663 cm^−1^. The weak band detected at 1655 cm^−1^ corresponds to the stretching vibrations of C=O in the carboxylic acid present in BDC. The peaks at 1585 cm^−1^ and 1399 cm^−1^ can be attributed to the asymmetric and symmetric stretching to carboxyl functional groups, respectively^25^. The peak at 746 cm^−1^ is because of the O–H bending and C–H bending (in-phase). On the other hand, the peak at 663 cm^−1^ is assigned to the Zr–O stretching^25^. The basic characteristic peaks of –COOH in functionalized CNTs can be observed at 1000–1300 cm^−1^ (C–O vibrational band) and the peak at 1740 cm^−1^ can be attributed to the stretching vibrations of the C=O bond of carbonyl or carboxyl groups present in GO that these peaks related to the carbonaceous materials only^24,30^. The results obtained from FTIR analysis indicated that the adsorption of MG and MB dyes onto nanoparticles could be due to π–π stacking interaction of aromatic structures of dye molecules with π systems on the surface of adsorbent and the aromatic ring in the structure of metal–organic frameworks. This interaction might be due to the electrostatic attraction, and hydrogen bonds between the oxygen-containing functional groups of adsorbent as well as the carbonyl functional groups of MR and the sulfone functional groups of MO, respectively^3,15,24^. Thus, the appearance of these characteristics’ peaks showed the successful synthesis of UiO-66 metal–organic frameworks and its composite nanoparticles.

**Figure 2:**
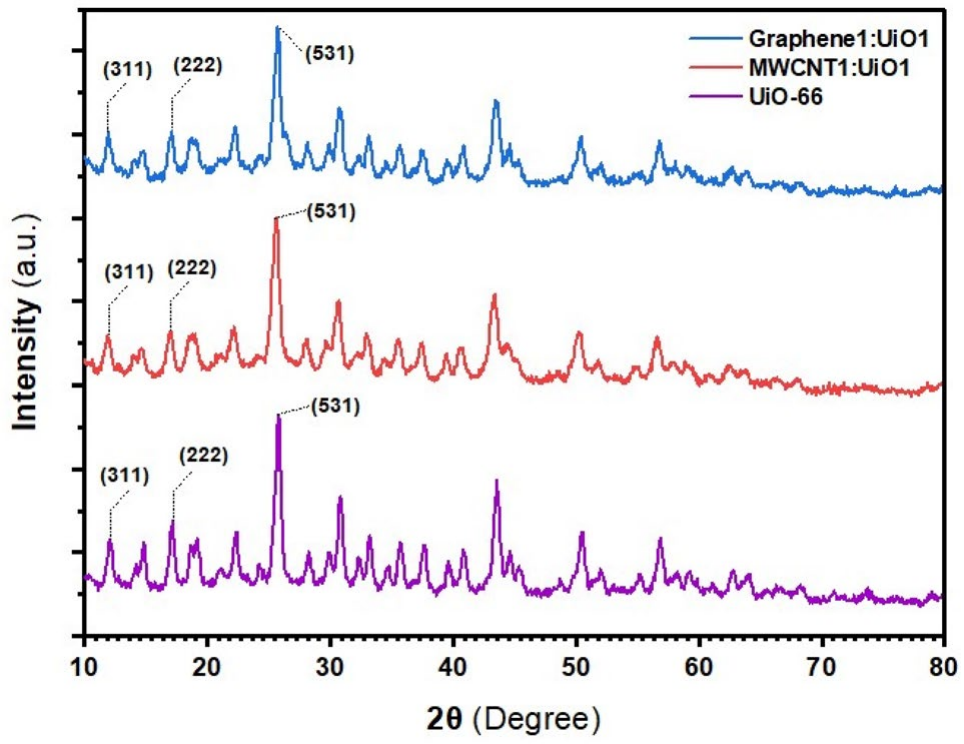
XRD patterns of UiO-66, MWCNT1:UiO1, and Graphene1:UiO1.

The XRD patterns of the UiO-66 and nanocomposites are revealed in Fig. 2. The major peaks were observed at 2θ values of 12.3°, 17.1° and 25.9°, corresponding to the (311), (222), and (531) crystal planes of UiO-66 material^1,9,23^. Similar to UiO-66, in the XRD spectrum of MWCNT1:UiO1 and Graphene1:UiO1, the characteristic peaks at 2θ of 12.3°, 17.1° and 25.9° have appeared, which indicate that these nanomaterials have been well synthesized and almost free of any impurity, but the peak intensity of the nanocomposites is lower than that of UiO-66. This can be attributed to the interactions between UiO-66 and carbonaceous materials i.e. CNTs-COOH as well as graphene oxide. The similar XRD patterns of the hybrid nanoparticle with UiO-66 indicate that MWCNTs-COOH and graphene oxide do not prevent the formation of crystalline structure of UiO-66.

**Figure 3:**
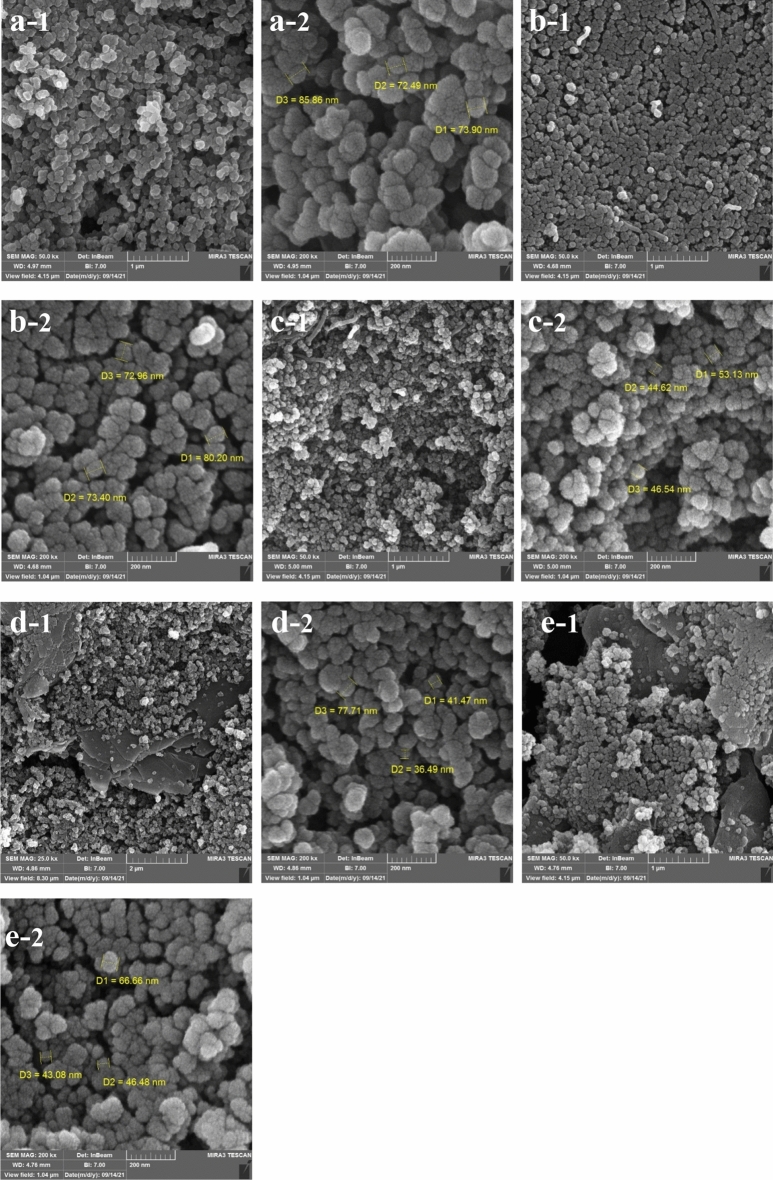
FESEM images of (**a**) UiO-66, (**b**) MWCNT1:UiO1, (**c**) MWCNT2:UiO1, (**d**) Graphene1:UiO1, (**e**) Graphene2:UiO1.

Figure 3 displays the morphology and structure of UiO-66 MOF and their composite nanoparticles. Obviously, the obtained images reveal round-shaped crystals morphology of UiO-66 nanoparticles with particle size of about 70–100 nm, which is similar to the results reported in the literature^24,30,31^. The particle size of the composites diminished with adding MWCNT-COOH and graphene oxide, and the composites nanoparticles became more irregular. This may be attributed to the coordination between the oxygen groups in the graphene oxide layers and Zr^4+^ mental center in the UiO-66, which prevented the aggregation of the UiO-66 crystallites and increased dispersion^30^.

The interactions of pure UiO-66 and carbonaceous samples with the dye molecules depend on the initial pH of dye solutions owing to the affection of pH on the adsorbent surface charges. Therefore, the initial pH of the dye solutions was presented in Table 1 to assess the effect of initial pH on adsorption capacities that it decreased gradually by increasing the pH for anionic MR and MO dyes^4^. Based on the previous studies, it was concluded that the nanocomposites have a positive zeta potential, which is suitable for the adsorption of anionic dyes, therefore, more capable of adsorbing more anionic dyes, while it is unfavorable to adsorb cationic dyes^24^.

**Table 1:** Initial pH of the dye solution.

Dye	Concentration		
	10 ppm	20 ppm	40 ppm
MR	4.31	3.84	3.61
MO	5.25	5.2	5.01
MB	4.15	4.1	3.95
MG	3.82	3.8	3.6

**Figure 4:**
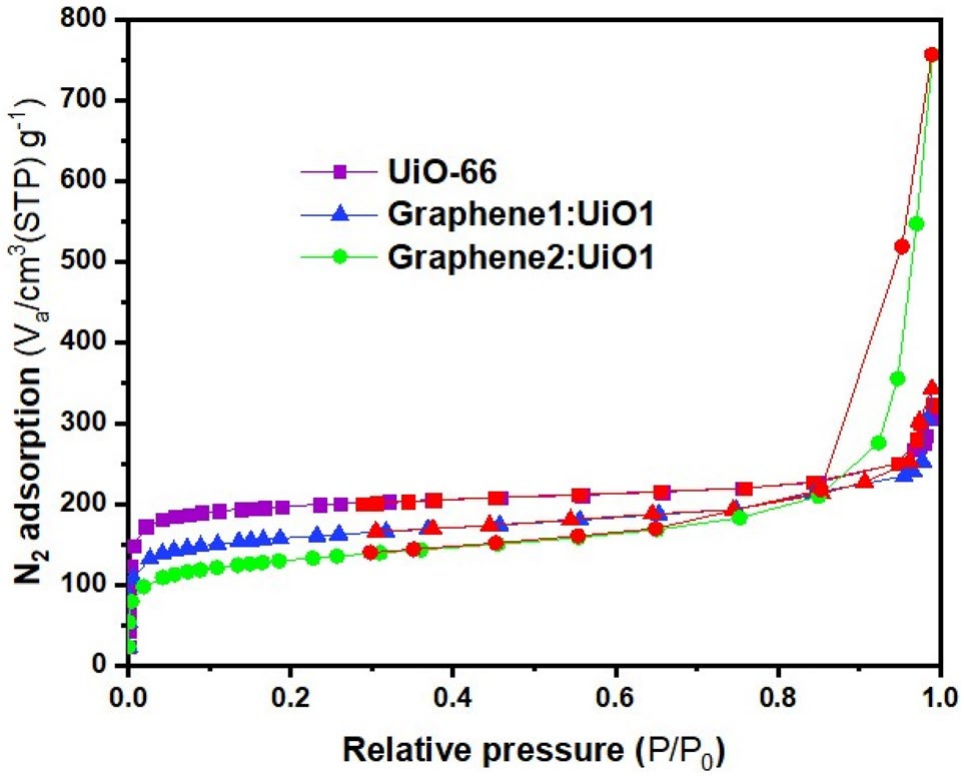
N_2_ adsorption/desorption isotherms (desorption shown with red symbols) of samples.

**Figure 5:**
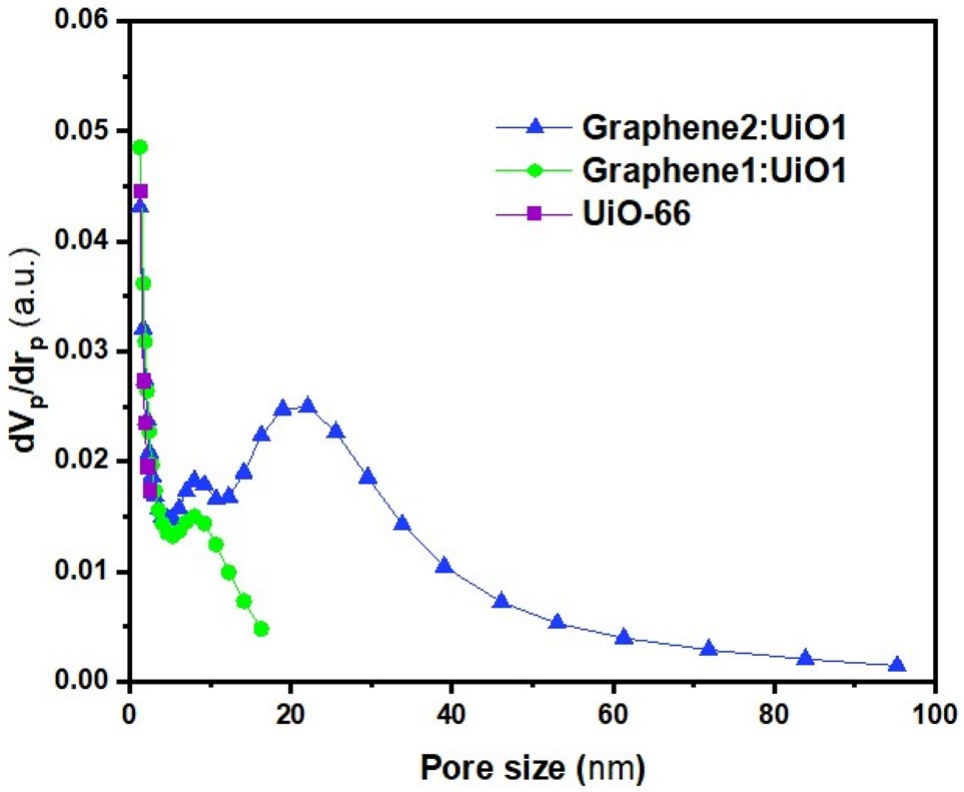
Pore size distributions of samples in this study.

The BET surface area, total pore volumes, and mean pore diameters of UiO-66 and its composites can be found in Table 2. The BET surface area of UiO-66 MOF is about 750.72 m^2^/g, which is within the range of values reported in the literature by other researchers^9,17,32^. The measured BET surface areas of Graphene1:UiO1 and Graphene2:UiO1 have been 578.93 m^2^/g and 466.3 m^2^/g, respectively. The BET surface area of the as-synthesized composite hybrid nanoparticle is lower than that of pure MOF, while the total pore volume and average pore diameter of as-synthesized composite are higher than those of UiO-66 because of formation of new pores, which is favorable for the adsorption of dye molecules^33^. Based on the results outlined in Table 2, it is clear that total pore volume and average pore diameter increased by elevating the concentration of graphene oxide because of the formation of additional porosity in the interfacial space between the UiO-66 and graphene oxide^34^. The porosity of the UiO-66 and its composites was investigated by N_2_ adsorption/desorption isotherms at 77 K, as depicted in Figure 4 with the pore size distribution displayed in Figure 5. According to Figs. 4 and 5 as well as Table 2 pure UiO-66 nanoparticles showed IUPAC reversible type-I isotherm and microporous structure (approximately 2 nm)^34,35^. Due to the presence of carbonaceous materials in the structure of this hybrid nanoparticle, the microporous structure of the nanoparticles changed to mesoporous. All nanocomposites possess a mesoporous structure (in range of 2–50 nm) and because of presence of mesopores in the MOFs, the hysteresis loop was appeared in the N_2_ adsorption/desorption isotherms^4^.

**Table 2:** Physical properties of adsorbents obtained from BET analysis.

Samples	S_BET_ (m^2^/g)	Total pore volume (cm^3^/g)	Average pore diameter (nm)
UiO-66	750.72	0.428	2.2804
Graphene1:UiO1	578.93	0.4252	2.9376
Graphene2:UiO1	466.3	1.0726	9.2008

EDS-MAP refers to methods used for studying the elemental compositions in nanoparticles. Figure S1a–c represents the EDAX results of the UiO-66 and their composite nanoparticles. The main elements in the adsorbent were Zr, C, and O; other peaks in the EDAX data corresponding to various elements indicated that the contaminates remained even after the washing process during the synthesis step. Figures S2, S3a–f reveal the EDS dot mapping for the MOF composite nanoparticles. The figures show that the elements had a uniform distribution over the surface of the adsorbents. As a result, it was concluded that the fabricated particles had highly porous structures which could be sufficient for the adsorption process.

Photoluminescence (PL) spectra for Graphene1:UiO1 nanoparticle at different excitation wavelengths is depicted in Fig. S4. This composite was investigated at different excitation wavelengths of 300, 350, 400, 450, 500, and 600 nm. When the excitation wavelength was gradually altered from 300 to 600 nm, the PL peaks shifted rapidly. It is worth mentioning that that after the excitation of 450 nm, the peaks did not shift considerably. The excitation-dependent PL characteristics of the different emission spectra are the result of the electron hole pairs due to the sp^2^ and the sp^3^ matrix of graphene in the structure.

### Adsorption kinetics of synthesized adsorbents

**Figure 6:**
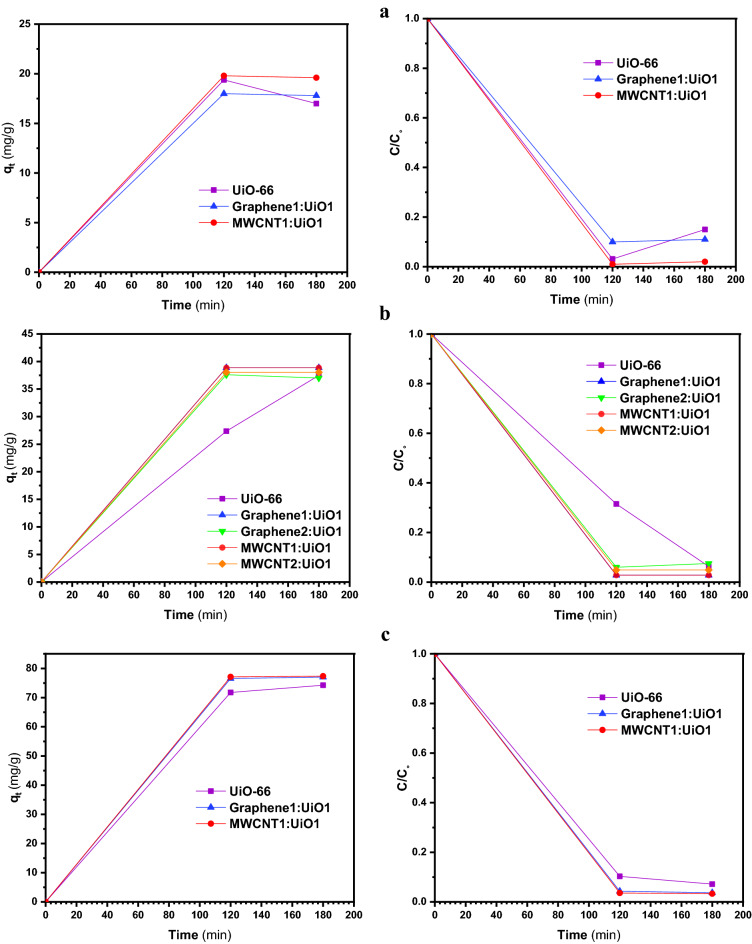
Adsorption capacity and solution concentration versus contact time of MR at different concentrations including (**a**) 10 ppm, (**b**) 20 ppm, (**c**) 40 ppm.

**Figure 7:**
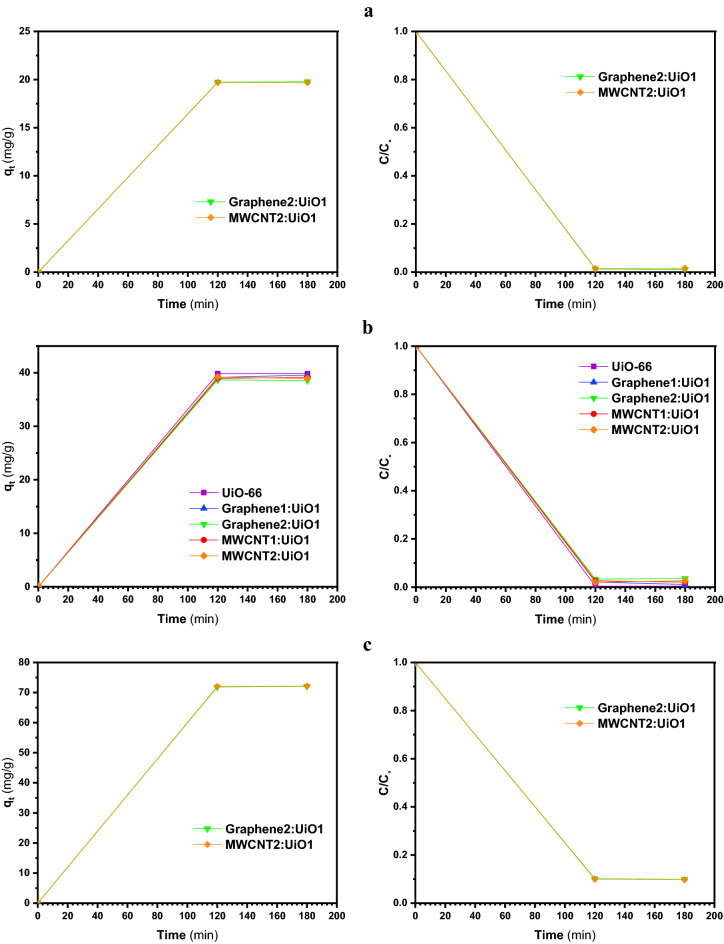
Adsorption capacity and solution concentration versus contact time for different MO initial concentrations of (**a**) 10 ppm, (**b**) 20 ppm, (**c**) 40 ppm.

**Figure 8:**
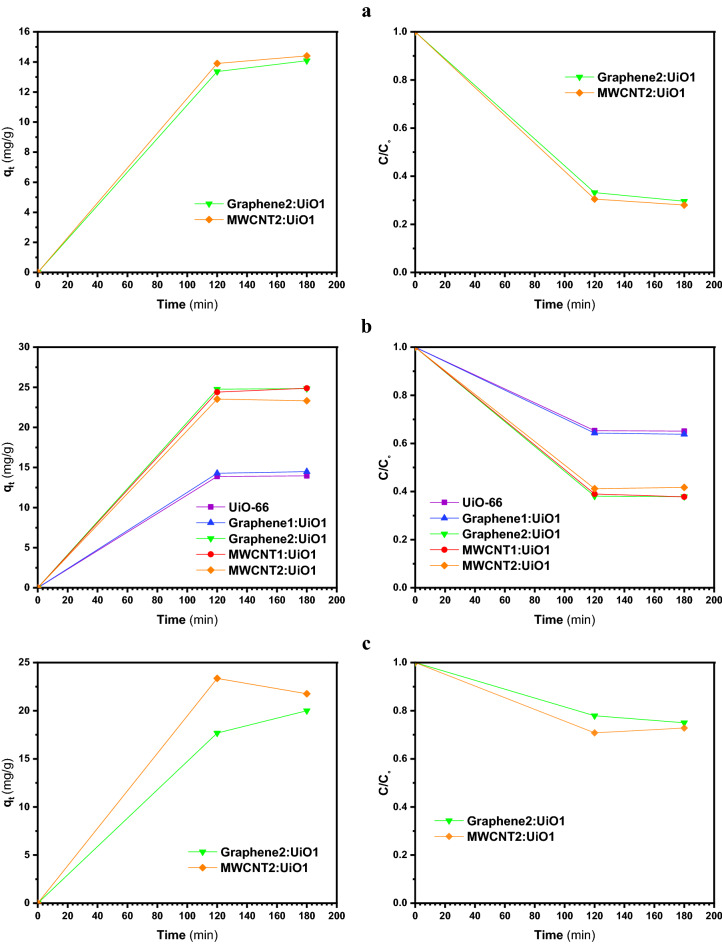
Adsorption capacity and solution concentration in various contact time of MB with initial concentrations of (**a**) 10 ppm, (**b**) 20 ppm, (**c**) 40 ppm.

**Figure 9:**
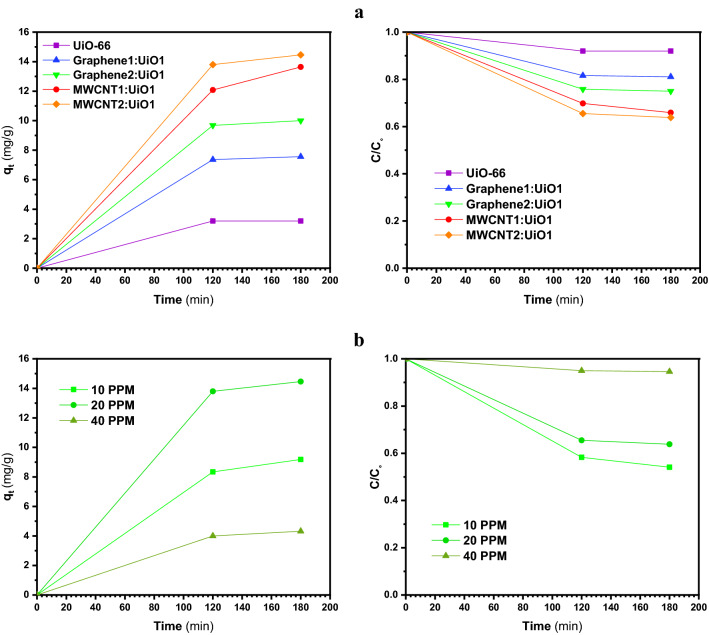
(**a**) Adsorption capacity and solution concentration versus contact time of MG 20 ppm, and (**b**) impact of MWCNT2:UiO1 on MG.

**Figure 10:**
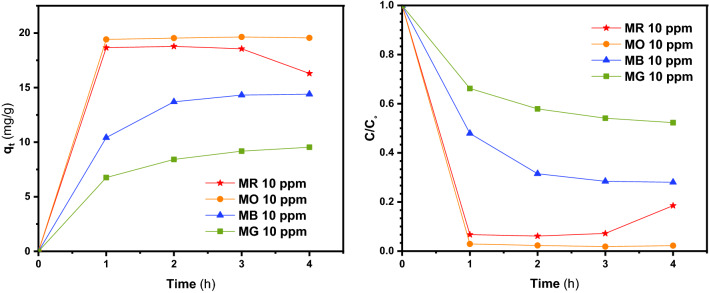
Stability tests of different dyes at concentration of 10 ppm for MWCNT:UiO.

Figures 6, 7, 8 and 9a display the experimental adsorption results and the impact of different synthesized adsorbents on different cationic and anionic dyes. Figure 9b reveals the impact of MWCNT2:UiO1 on cationic malachite green (MG) dye with different initial concentrations as 10 mg/L, 20 mg/L, 40 mg/L. Obviously, the adsorption capacity for both cationic and anionic dyes reached equilibrium after about 2 h of the experiment. Following the studies conducted by researchers, it is deduced, the equilibrium time of 2 h is selected for some of adsorption processes^3,23^. According to the experimental adsorption results, it is obvious, the synthesized adsorbents showed the highest adsorption efficiency towards MR and MO dyes and MWCNT:UiO indicated higher adsorption capacity towards MG and MB cationic dyes compared with single UiO-66. It can also be found from Fig. 6b, the concentration of red solution of MR has dropped from 20 to 0.57 mg/L after 2 h in the presence of MWCNT:UiO and the adsorption capacity increased initially. This phenomenon is owing to the presence of many empty sites to adsorb dye molecules at the beginning of the reaction and then slowed down^3^. On the other hand, as indicated in Fig. 9 the concentration of MG solution diminished from 20 to 13.1 mg/L after 2 h. This clearly shows that MWCNT:UiO has a higher ability to adsorb anionic dyes than cationic dyes. Figure 10 displays the stability tests for adsorption of dyes onto MWCNT:UiO (both cationic and anionic dyes) from distilled water with an initial concentration of 10 mg/L. The stability tests were performed to supplement existing tests until identifying the effect of time on adsorption process. Experimental adsorption results indicated that adsorption capacity of the materials with time were stable up to 4 h toward both cationic and anionic dyes as well as the amount of dye uptake on the adsorbent increased with time.

### Adsorption mechanism

To assess the dye adsorption mechanism of pure UiO-66 and its composite nanoparticles toward anionic and cationic dyes, FTIR analysis is performed on dyes, synthesized adsorbent before dyes adsorption. Following the studies conducted by researchers, it is deduced, the results obtained from FTIR analysis indicated that the adsorption of MG and MB dyes onto nanoparticles could be due to π–π stacking interaction of aromatic structures of dye molecules with π systems on the surface of adsorbent and the aromatic ring in the structure of metal–organic frameworks. This interaction might be due to the electrostatic attraction, and hydrogen bonds between the oxygen-containing functional groups of the adsorbent as well as the carbonyl functional groups of MR and the sulfone functional groups of MO, respectively^3,15,24^.

### Adsorption isotherm

**Figure 11:**
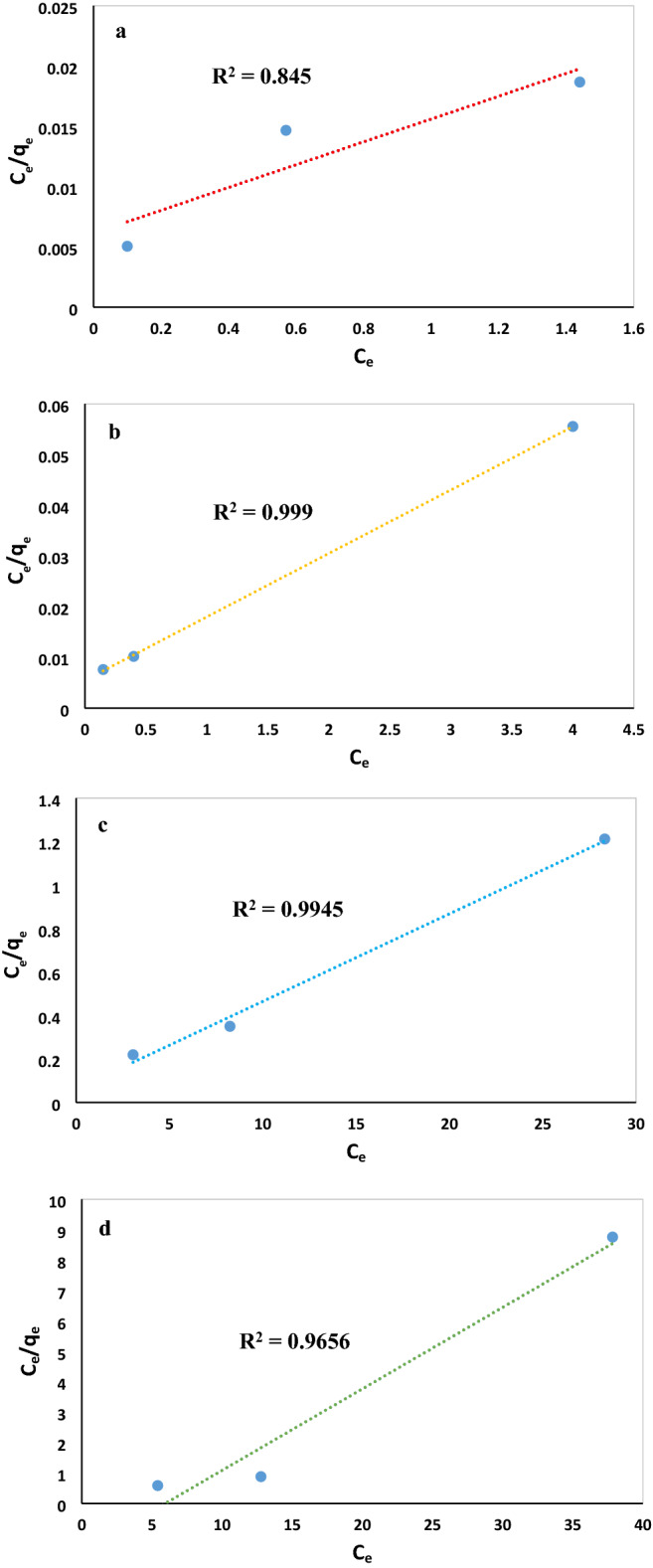
Langmuir isotherm fitting for (**a**) MR, (**b**) MO, (**c**) MB, (**d**) MG on MWCNT:UiO.

**Figure 12:**
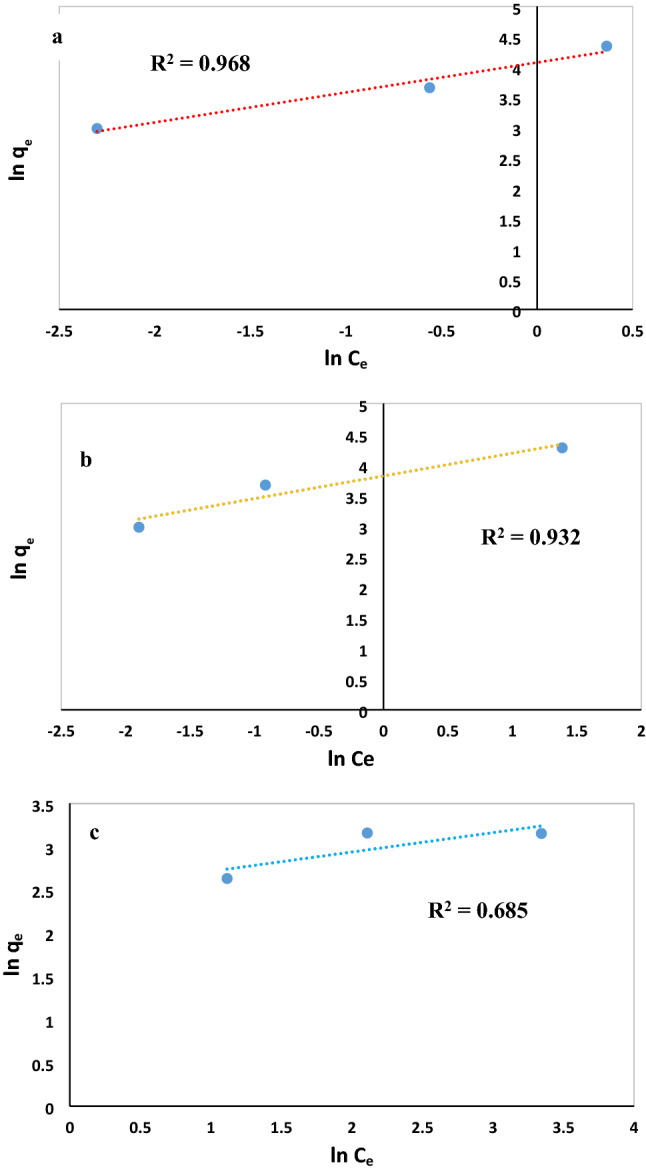
Models fit of Freundlich isotherms for (**a**) MR, (**b**) MO, (**c**) MB over MWCNT:UiO.

The adsorption isotherms for anionic dyes and cationic dyes were investigated at various initial dye concentrations to find the mechanism of the adsorption, tendency and surface properties of adsorbent and to determine the maximum uptake of various dyes for MWCNT:UiO. Also, two isotherm models of Freundlich and Langmuir models were examined in this study with the results depicted in Figs. 11a–d, 12a–c, respectively. The Langmuir adsorption model assumes that monolayer adsorption takes place onto a specific homogenous site in the surface of the adsorbent. Also, all active points have the same energies of adsorption without immigration of adsorbate molecules across the plane of the surface. However, Freundlich isotherm is based on the multilayer adsorption onto the surface of adsorbents with heterogeneous energies. Linear forms of Langmuir and Freundlich models are expressed in the following equations respectively:

3 $$\frac{{{\text{C}}}_{{\text{e}}}}{{{\text{q}}}_{{\text{e}}}}= \frac{1}{{{\text{K}}}_{{\text{L}}}{{\text{q}}}_{{\text{max}}}}+ \frac{{{\text{C}}}_{{\text{e}}}}{{{\text{q}}}_{{\text{max}}}}$$

4 $${{\text{ln q}}}_{{\text{e}}}={{\text{ln K}}}_{{\text{F}}}+ \frac{1}{{\text{n}}}{{\text{ln C}}}_{{\text{e}}}$$

where q_e_ (mg/g) and C_e_ (mg/L) represent the equilibrium adsorption capacity and equilibrium concentration of the dye, respectively, q_max_ (mg/g) shows the theoretical maximum adsorption capacity, and K_L_ (L/mg) is the Langmuir binding constant related to the rate of adsorption. The K_F_ and n_F_ are Freundlich constants that indicate adsorption capacity and adsorption intensity, respectively. If the value of n is larger than 1 for a dye, it means that it is more possible to be adsorbed onto the adsorbent. The Freundlich isotherm constant (n) for MR, MO, and MB is higher than 1, indicating that the adsorption of these dyes onto MWCNT:UiO is suitable. Based on the results outlined in Table 3, the MR, MO, and MB adsorption isotherms were fitted with the Langmuir and Freundlich model and MWCNT:UiO exhibited a maximum adsorption capacity of 105.26 mg/g for MR, while the MG isotherm is in agreement with the Langmuir model. The results of Table 4 show the MWCNT:UiO nanocomposite exhibited the perfect efficiency for adsorption of MR anionic dye from distilled water regarding the maximum adsorption capacity. Obviously, the maximum adsorption capacity of MWCNT:UiO is higher than that of most adsorbent materials reported previously^24,36–39^.

**Table 3:** The isotherms constants of adsorption models for various dyes.

Dye	Langmuir			Freundlich		
	q_max_ (mg/g)	K_L_	R^2^	K_F_	n	R^2^
MR	105.26	1.557	0.845	58.84	2.02	0.968
MO	80	2.272	0.999	45.51	2.69	0.932
MB	24.81	6.52 × 10^–1^	0.994	12.054	4.464	0.685
MG	3.73	1.68 × 10^–1^	0.965	–	–	–

**Table 4:** Comparison of obtained experimental results for adsorption of MR dye on MWCNT1:UiO1 nanoparticle.

Adsorbent	Adsorption capacity (mg/g)	References
Thiosemicarbazide modified chitosan	17.31	Mozaffari et al.^36^
Clinoptilolite	26.0	Ioannou et al.^37^
Active carbon	40.44	Santhi et al.^38^
Guargum powder	66.7	Saxena and Sharma^39^
CNTs-COOH	83.4	Sang et al.^24^
MWCNT:UiO nanoparticle	105.26	This work

## Conclusions

In this investigation, hybrid nanocomposites of carbonaceous materials that include carboxylic acid functionalized CNTs (MWCNTs-COOH) and graphene oxide (GO) over UiO-66 with different molar ratios (2.9 and 5.8 wt.%) were successfully synthesized through as solvothermal method for the removal of four different cationic and anionic dyes from distilled water. The amounts of carbonaceous materials added in the preparation of the composites were separately 1.5, 2.9, and 5.8 wt.% of the initial material weight. The properties of synthesized adsorbents were characterized via XRD, FTIR, FESEM, PL, BET surface area analysis, and EDS-MAP. Adsorption experiments indicated that composite nanoparticles synthesized by carboxylic acid functionalized CNTs (MWCNTs-COOH) and graphene oxide (GO) exhibited higher adsorption efficiency towards MG and MB cationic dyes compared with single UiO-66. Also, the performance of these composite nanoparticles improved by elevating the concentration of carbonaceous materials and displayed maximum improvement for MWCNT2:UiO1 nanocomposites. Furthermore, experimental adsorption results showed that the synthesized adsorbents exhibited the highest adsorption efficiency towards MR and MO dyes and could adsorb 98% of MR and MO, as well as 72% of MB and 46% of MG. The FTIR analysis demonstrated that the possible reasons for the high adsorption could be owing to π–π stacking interaction of aromatic structures of dye molecules with π systems on the surface of adsorbent, electrostatic attraction, and hydrogen bonds between the oxygen-containing functional groups of adsorbent, the carbonyl functional groups of MR, and the sulfone functional groups of MO, respectively. Generally, the hybrid nanoparticles revealed higher adsorption capacity for anionic dyes compared to cationic dyes and represented great usage potential for water treatment.

## Supplementary Information

Supplementary Figures.

## Data Availability

All data generated or analysed during this study are included in this published article (and its Supplementary Information files).
