# Supplementary material for: Adsorption of different anionic and cationic dyes by hybrid nanocomposites of carbon nanotube and graphene materials over UiO-66
Source: Sci Rep. 2022 Nov 27;12:20415. doi: 10.1038/s41598-022-24891-2 (PMC9701811; doi:10.1038/s41598-022-24891-2)
Supplement: Supplementary file 1 — Supplementary Figures. [file 41598_2022_24891_MOESM1_ESM.docx]

**Supporting Information**

**Adsorption of** **different anionic and cationic dyes by hybrid nanocomposites of carbon nanotube and graphene materials over UiO-66**

Mohammadreza Athari ^a^, Moslem Fattahi ^a,^*, Mohammadreza Khosravi-Nikou^b^, Aliasghar Hajhariri^c^

^a^ Department of Chemical Engineering, Abadan Faculty of Petroleum Engineering, Petroleum University of Technology, Abadan, Iran

^b^ Department of Gas Engineering, Ahvaz Faculty of Petroleum, Petroleum University of Technology, Ahvaz, Iran

^c^ Fakultät für Verfahrens- und Systemtechnik (FVST), Otto von Guericke Universität (OVGU), Magdeburg, Germany

*Corresponding author: fattahi@put.ac.ir

**b**

**a**

**c**

Figure S1: EDAX elemental composition of (a) UiO-66 (b) Graphene1:UiO1 (c) MWCNT:UiO1

| 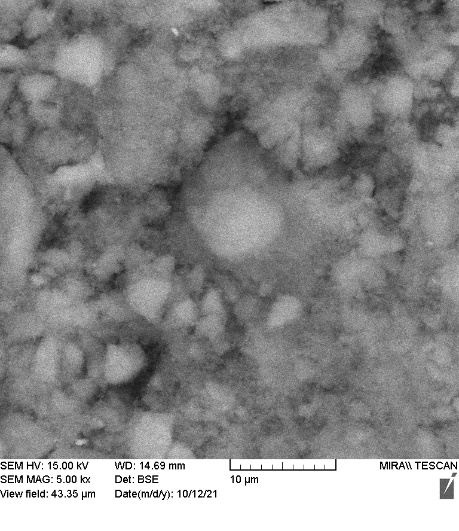  **a** **b** **c**    **d** **e** **f**  |
| --- |

Figure S2: EDS dot mapping of Graphene1:UiO1: (a) overall, (b) Zirconium, (c) carbon, (d) oxygen, (e) chlorine (f) silicon

| ***a*** ***b*** ***c*** 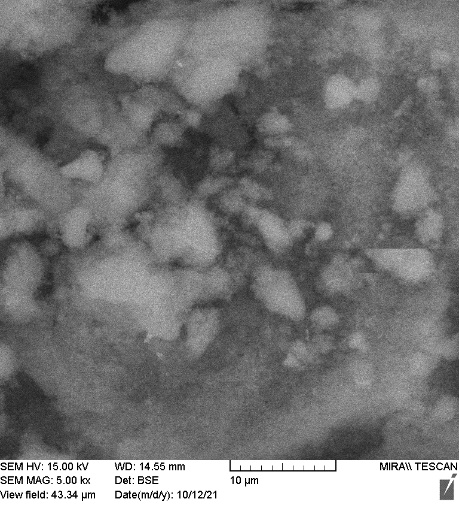    ***d*** ***e*** ***f*** |
| --- |

Figure S3: EDS dot mapping of MWCNT:UiO1: (a) overall, (b) zirconium, (c) carbon, (d) oxygen, (e) chlorine (f) silicon

Figure S4: Photoluminescence (PL) spectra of Graphene1:UiO1 at various excitation wavelengths
